# Changes in Lipid and Fatty Acid Composition During Intramacrophagic Transformation of *Leishmania donovani* Complex Promastigotes into Amastigotes

**DOI:** 10.1007/s11745-017-4233-6

**Published:** 2017-02-04

**Authors:** Hana Bouazizi-Ben Messaoud, Marion Guichard, Philippe Lawton, Isabelle Delton, Samira Azzouz-Maache

**Affiliations:** 1 Institut de recherche pour le développement (IRD), UMR InterTryp IRD/CIRAD, campus international de Baillarguet, Montpellier, France; 2 Department of Parasitology and Medical Mycology, Lyon University, Lyon, France; 3 Inserm U1060 CarMeN Laboratory, INSA-Lyon, Villeurbanne, France

**Keywords:** *Leishmania donovani* complex, Amastigotes, Lipid variations, Cholesterol, Fatty acids, Phospholipids

## Abstract

*Leishmania* sp., are trypanosomatid parasites that are phagocytized by human and animal macrophages. Transformation from the vector promastigote stage to the intracellular amastigote host cell stage is mandatory, since development in the host depends on the internalization of the parasite. We identified and analyzed the lipids involved in the promastigote to amastigote transformation process in the *Leishmania donovani* complex. Four lipid classes, phospholipids, free fatty acids, triglycerides and sterols were studied. The derivatization method of Bligh and Dyer was used to establish the fatty acid composition in each stage of the parasite. To stay within the context of *Leishmania* infection, we used amastigotes extracted from macrophages after experimental *in vitro* infection. The purification process was checked by electronic microscopy, the absence of major contamination by host-cell debris and a correct purification yield validated our experimental model. Our results show that free fatty acids and cholesterol increased, whereas triglycerides and ergosterol decreased during the transition between promastigotes to amastigotes. With respect to phospholipid classes, we found increased proportion of sphingomyelin and phosphatidylserine and lowered proportion of phosphatidylinositol and lysophosphatidylethanolamine. Regarding fatty acid composition, a significant increase of n-7 fatty acids was observed in amastigotes. Overall, the total n-6 fatty acids were decreased in PL. Several of the changes were also observed in TG and free fatty acids. Particularly, n-7 fatty acids and 20:4n-6 were highly increased, whereas n-9 fatty acid and n-6 precursors decreased.

## Introduction

*Leishmania donovani* and *L. infantum* are the causative agents of visceral leishmaniasis (VL) in humans and of canine leishmaniasis in dogs. *Leishmania* is an intracellular pathogen, whose establishment depends on its successful internalization and multiplication inside macrophages, its mammalian host cell. The *Leishmania* life cycle is divided into two phases, each of them involving a different stage, the promastigote stage in the insect vector and the amastigote stage inside the host’s macrophages. The promastigotes inoculated during the blood meal of the hematophagous sandfly are phagocytosed by endocytosis and undergo a transformation into amastigotes within a parasitophorous vacuole of phagolysosomal origin. This process is a vital step in the *Leishmania* life cycle and could be pivotal in the research for new treatments against *Leishmania*.

To date, studies have essentially focused on the role of surface glycosylated residues and proteins in host-parasite interactions. Recent interest has grown for the role of lipids in Trypanosomatids and especially in *Trypanosoma cruzi*, *T. brucei* and *Leishmania* spp. [1]. Lipid metabolism is of paramount importance for parasites, and especially for intracellular parasites, which rely on a complex system of uptake and synthesis mechanisms to satisfy their lipid needs.

The parameters of this system change dramatically as the parasite transits through the various stages of its life cycle. Within host cells, intracellular pathogens often develop in specialized vacuoles and the flow of lipids between host and pathogen-controlled membranous compartments is pivotal to the pathogen’s ultimate success [2–4]. Intracellular pathogens have evolved sophisticated mechanisms to manipulate and tap into the lipid metabolism of their host cells. These include interference with vesicular and non-vesicular cellular lipid trafficking in viral [5], bacterial [6] and protozoal [7] pathogens. According to Zhang and Beverley [8], phospholipids (PL) and sphingolipids are both abundant and critical to virulence and viability in *Leishmania*. The success of miltefosine as an orally available antileishmanial drug is an important validation of lipid metabolism as a drug target. Moreover, it has been previously reported that macrophage cholesterol is important for parasite internalization. The entry of intracellular parasites and particularly of *Leishmania*, involves interaction with the plasma membrane of host cells. A number of previous studies have demonstrated the requirement of membrane cholesterol in host-pathogen interactions [9, 10]. Cholesterol (C) is an important component of higher eukaryotic cellular membranes and plays a crucial role in the function and the organization of membrane proteins and receptors [11, 12], some of which being necessary for parasite entry [4].

Our goal was to analyze the variations in major lipid classes during the metamorphosis from the promastigote to the amastigote stage, in terms of quantities and fatty acid composition. Biological membranes are composed of fatty acids (FA) and phospholipids, which are present in constant proportions, but when exposed to some stress conditions, such as pathogens or drugs, these proportions might change [13]. The identification of these specific variations could be used as biomarkers to study virulence or resistance to treatments currently used against leishmaniasis.

## Materials and Methods

### Promastigote Culture

The strains used in this study were *L. donovani* and *L. infantum* that both belong to the *Leishmania donovani* complex. *Leishmania donovani* (LCR-133) was provided from the *Leishmania* Reference Center, Jerusalem, Israel and the *L infantum* strain was isolated in Spain from an infected dog. The promastigotes were grown at 22 °C in TC-199 medium (GE Healthcare, Pasching, Austria) supplemented with 20% fetal bovine serum and 2% antibiotics (streptomycin and penicillin, GE Healthcare, Pasching, Austria). The parasites were cultured in 75-cm^2^ culture flasks and were isolated during their exponential growth phase by centrifugation at 1300×*g* for 15 min. The flasks containing the parasites were washed 3 times with 10 ml PBS and before lipid analysis, a total protein assay was performed with the Bradford method.

### Macrophage Culture

The BALB/c mice cell line J774A.1 (European Collection of Cell Cultures) were maintained at 37 °C with 5% CO_2_ by successive passages in RPMI-1640 culture medium (PAA) containing 10% (v/v) fetal bovine serum (Sigma, St-Quentin Fallavier, France), 1% MycoKill AB (GE healthcare, Pasching, Austria) and 1.5% Penicillin/Streptomycin (PAA, GE Healthcare, Pasching, Austria).

### Infection of Macrophages and Isolation of Amastigotes

The macrophages from five 75-cm^2^ flasks were infected at a ratio of 10 parasites per cell. After 72 h, the infected cells were collected by trypsinization, centrifuged at 300×*g* for 5 min and then suspended in PBS. The infected cells were disrupted by repeated passaging—at least 30—through a syringe fitted with a 22-gauge needle (Sigma-Aldrich, St-Quentin Fallavier, France). To facilitate the process, the cells were weakened by one freeze–thaw cycle at −80 °C. Several centrifugations at 300×*g* for 5 min were performed to eliminate host-cells debris. The amastigote-containing suspension was then filtered through a 3-µm polycarbonate membrane (Merck, Molsheim, France) to remove the remaining host-cell debris. The purified amastigotes were concentrated by centrifugation at 2000×*g* for 10 min.

The infected macrophages were Giemsa-stained, the intracellular amastigotes were counted, and after isolation from macrophages, the number of isolated amastigotes was evaluated, after Giemsa staining, to calculate the purification yield. Various parameters, such as the number of freeze–thaw cycles and the number of disruption cycles were optimized to improve the purification yield.

For each sample, a total protein assay was performed with the Bradford method and 1 mg of total extract was used for lipid analysis.

### Transmission Electron Microscopy (TEM)

Axenic and purified intracellular amastigotes were fixed with 4% glutaraldehyde in 0.2 M cacodylate buffer, pH 7.4, post-fixed with 1% osmium tetroxide and 0.8% potassium ferrocyanide in 0.1 M cacodylate buffer, dehydrated in a graded series of acetone and embedded in Epon. Ultrathin sections were stained with uranyl acetate and lead citrate and observed under a Jeol 1400 JEM transmission electron microscope (Tokyo, Japan) set at 120 kV.

### Lipid Analyses

#### Total phospholipids, triglycerides and free fatty acids

Total lipids were extracted from cell lysates (0.1% Triton in water) by the method of Bligh and Dyer [14] after addition of butylated hydroxytoluene (BHT, 50 µM final) as an antioxidant. PC17:0, 17:0, TG17:0 (Sigma, St-Quentin Fallavier, France), were added to serve as internal standards. Lipids were separated on thin layer chromatography (silica gel 60 plates, Merck, Fontenay sous Bois, France) using the solvent system hexane/diethylether/acetic acid 80:20:1 (v/v), revealed under UV light after spraying 0.05% 2′7′-dichlorofluorescein (Sigma) in methanol and identified by comparison with an authentic standard spotted on the same plate. Phospholipids, free fatty acids and triglycerides (TG) spots were scrapped from the plate and transmethylated using boron trifluoride (Sigma, St-Quentin Fallavier, France). The resulting fatty acid methyl esters (FAME) were analyzed by GC using a Hewlett-Packard system equipped with a Supelco SP2380 capillary column (60 m × 0.22 mm) with helium as a carrier gas. FAME were identified by comparison with commercial standards [15]. Results are expressed as molar percentage of each individual fatty acid calculated from the sum of all detected fatty acids taken as 100%. Quantification of PL, TG and free FA pools was made in correspondence with their fatty acid content using the internal standards.

#### Sterols

Lipid standards (cholesterol, epicoprostanol and ergosterol) and chemicals of the highest grade available were purchased from Sigma Aldrich (St-Quentin Fallavier, France). Mass spectrometry quality grade solvents were purchased from Fischer Scientific (Illkirch, France). Total lipids were extracted from cell pellets (1 mg) according to the method of Bligh and Dyer [14]. Organic phases corresponding to 100 µg of extract were evaporated to dryness under vacuum. Dried lipids were hydrolyzed for 45 min at 56 °C with potassium hydroxide 10 M in ethanol containing 2 µg of epicoprostanol used as internal standard. Sterols were extracted by hexane and water. After evaporation, sterols were derivatized with 100 µL of a mixture of bis(trimethylsilyl)trifluoroacetamide/trimethylchlorosilane 4/1 v/v for 1 h at 80 °C. Sterols, derivatized as trimethylsilyl-ethers, were analyzed by GCMS in a 7890A Gas chromatograph connected to a 5975C Mass Selective Detector (Agilent Technologies). Separation was achieved on a HP-5MS 30 m × 250 µm column (Agilent Technologies) using helium as carrier gas. The MSD was set up as follow: EI at 70 eV mode, source temperature at 230 °C. Data were acquired in SIM mode using 363.3, 368.3 and 370.3 quantitation ions (*m*/*z*) for ergosterol, cholesterol and epicoprostanol respectively. A calibration curve was obtained with cholesterol (1–8 µg) and ergosterol (1–16 µg) standards using the same method used for samples.

#### Phospholipids Analysis

Phospholipids (PL) were first separated by liquid chromatography under HILIC conditions and then detected by a Corona™ ultra RS Charged Aerosol Detector™ (CCAD, Thermo Scientific, USA). High performance liquid chromatography was performed using a Dionex UltiMate™ 3000 LC pump from Thermo Scientific (USA) equipped with a dual-gradient pump which allows a post-separation inverse gradient approach to compensate solvent gradient effects in CCAD detection. Separation of PtdCho, PtdEtn, PtdIns, PtdSer, CerPCho, LPtdCho, and LPtdEtn standards was achieved under HILIC conditions using a Thermo Accucore column (150 × 2.1 mm, 2.6 µm, Hilic, Thermo Scientific, USA). The mobile phase consisted of a gradient of (A) ACN/H_2_O (95:5, v/v) and (B) ACN/H_2_O (50:50, v/v) containing 10 mM ammonium acetate. The flow rate was at 800 µL·min^−1^. The injection volume was 5 µL and the column was maintained at 40 °C. Eluate from the HPLC system was introduced in a CCAD for detection of the phospholipids. The liquid chromatography and the CCAD were controlled by Chromeleon 7.2 (Thermo Scientific Dionex).

## Results

### Purification of Amastigotes

**Fig. 1:**
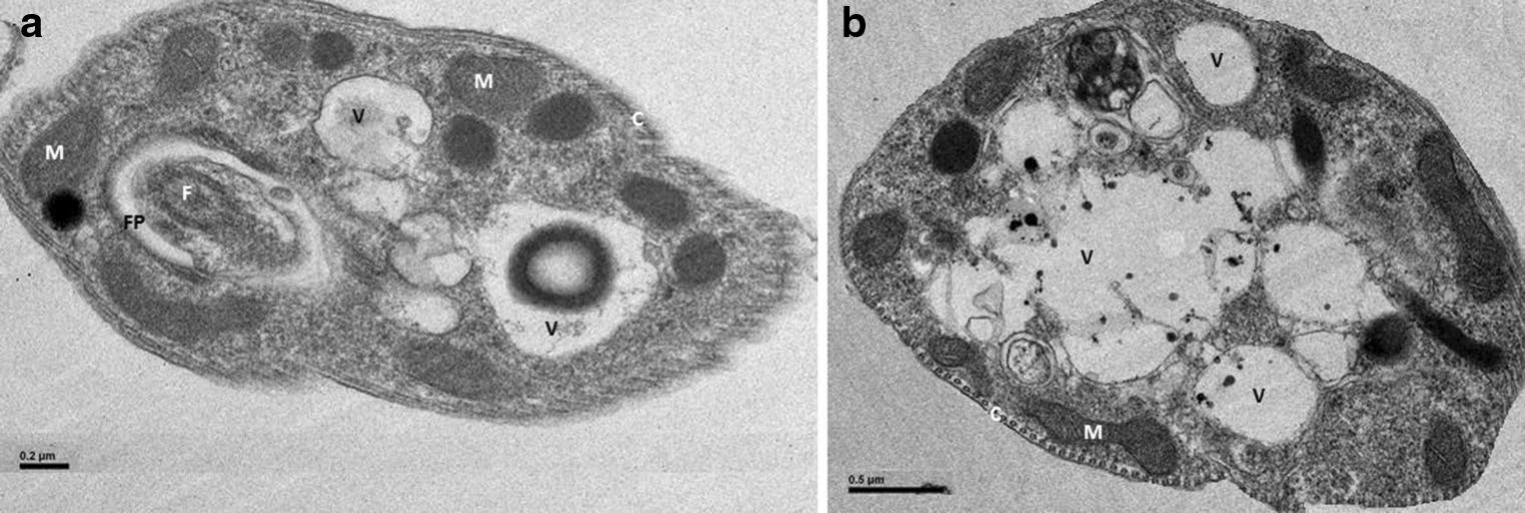
Electron microscopy of purified intracellular and axenic *Leishmania* amastigotes. **a** purified intracellular amastigotes of *L. donovani*, **b** purified intracellular amastigotes of *L. infantum*, the presence of vacuoles (V) in purified amastigotes could be explained by the observed cholesterol increase. *F* flagellum, *K* kinetoplast, *N* nucleus, *M* mitochondrion, *FP* flagellar pocket, *C* cytoskeleton

After standardization of our intra-macrophagic amastigotes purification method, the best results were obtained after 18 cycles of passaging of the cells by a Hamilton syringe, and after a short freezing—1 h—of the infected macrophages. The purification yield of amastigotes recovered from freeze-thawed cells (87.2%) was higher than when the cells were directly processed (45.6%). The purification process was monitored by optical (results not shown) and electron microscopy that showed that the intramacrophagic amastigotes obtained by our purification method had a regular shape and were in overall good condition. Electron microscopy also evidenced the absence of contamination by host-cell material at the end of the purification process (Fig. 1).

### Lipid Profiles

Independent cultures of *L. donovani* and *L. infantum* were prepared and processed for lipid analyses.

**Fig. 2:**
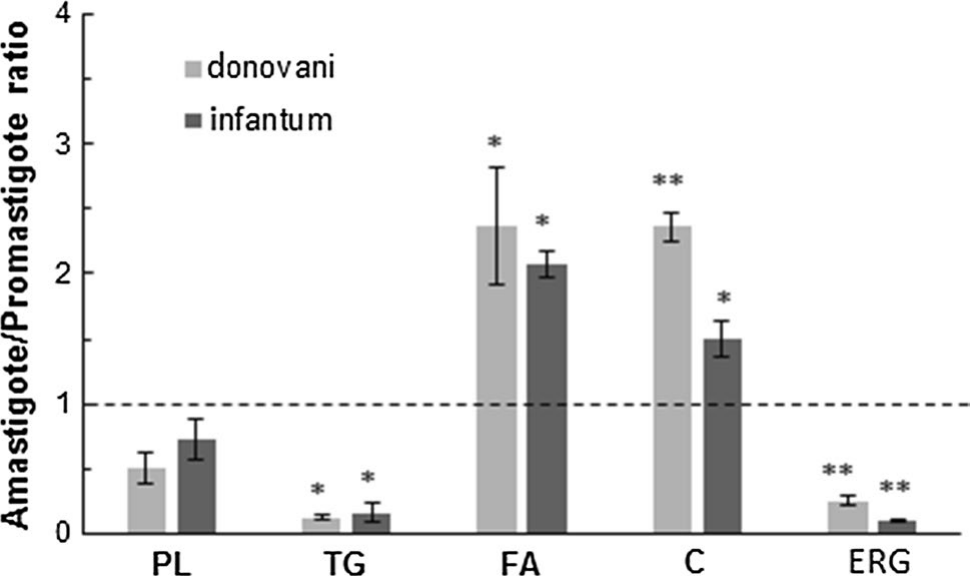
Lipid modifications in amastigotes. Lipid quantification was made by GC as detailed in materials and methods. The bars represent the ratio amastigote/promastigote for lipid quantities calculated as nmol/mg protein. Data are the means ± SD of 3 independent determinations. *PL* phospholipids, *TG* triacylglycerols, *FA* free fatty acids, *C* cholesterol, *ERG* ergosterol. *p ≤ 0.05, **p ≤ 0.01, significant differences between promastigote and amastigote by *t* test

We first determined the lipid distribution of major lipid classes (total PL, TG, free FA and sterols) in both parasite forms in order to highlight specific changes during differentiation of promastigote to amastigotes (Fig. 2). Both *Leishmania* species showed similar changes. Among the major differences in amastigotes compared to promastigotes was the marked increase of the free fatty acid pools by about 2-fold. By contrast, the PL content was decreased nearly by half without reaching statistical significance and TG were reduced by 85% percent. Regarding sterols, as expected, promastigote forms contained more ergosterol than cholesterol with an ergosterol/cholesterol ratio of about 2.5 (ergosterol 210 ± 18 nmol/mg protein and 355 ± 23 nmol/mg protein in *L. donovani* and *L. infantum*, respectively; cholesterol 83 ± 7 nmol/mg protein and 143 ± 21 nmol/mg protein in *L. donovani* and *L. infantum*, respectively). Of interest, the cholesterol content in amastigotes increased by nearly 2-fold in both species, whereas that of ergosterol decreased by about 80%, so that the ergosterol/cholesterol ratio lowered to less than 0.25.

**Fig. 3:**
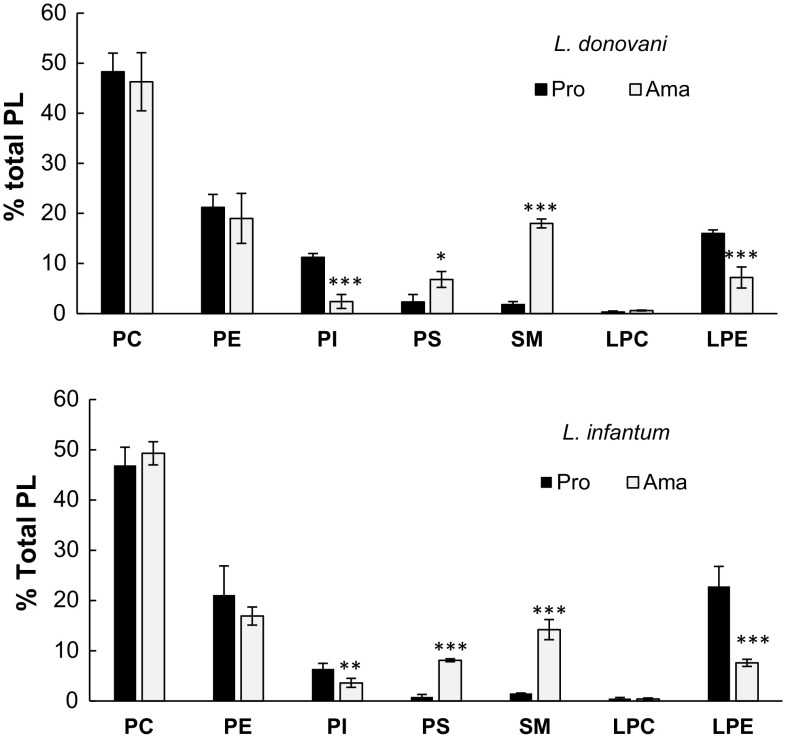
PL distribution in promastigote and amastigote. Lipid quantification was made by HPLC-CAD as detailed in materials and methods. The bars represent the % of total PL. Data are the means ± SD of four independent determinations. *PtdCho* phosphatidylcholine, *PtdEtn* phosphatidylethanolamine, *PtdIns* phosphatidylinositol, *PtdSer* phosphatidylserine, *CerPCho* Sphingomyelin, *LPtdCho* lysophosphatidylcholine, *LPtdEtn* lysophosphatidylethanolamine. *p ≤ 0.05, **p ≤ 0.01, ***p ≤ 0.001 significant differences between promastigote

Figure 3 shows the PL profiles. Again, very close data were found for both *Leishmania* species. In promastigotes, the predominant PL were PtdCho, PtdEtn and LPtdEtn. PtdIns, PtdSer, CerPCho and LPtdCho were found in much lower proportion. Other PL such as cardiolipin, phosphatidylglycerol and phosphatidic acid were not detected or at only trace amounts. Among the major changes in amastigotes were the marked increase of CerPCho and PtdSer. Conversely, LPtdEtn and PtdIns were significantly decreased. There was no change for major PL, PtdCho and PtdEtn.

### Fatty Acid Composition

Tables 1 and 2 show the fatty acid composition of major lipids, total phospholipids and triacylglycerols as well as free fatty acid profiles. Similar observations could be made for both *Leishmania* species.

**Table 1:** Fatty acid composition of total phospholipids, triacylglycerols and free fatty acid profile in *L. infantum* promastigote and amastigote

	Phospholipids		Triacylglycerols		Free fatty acids	
	Pro	Ama	Pro	Ama	Pro	Ama
14:0	0.4 ± 0.3	3.2 ± 0.3*	2.2 ± 0.7	2.8 ± 0.7	1.5 ± 1.2	5.7 ± 1.2**
16:0	21.3 ± 3.2	28.4 ± 0.9	5.8 ± 0.6	10.8 ± 0.8**	6.4 ± 1.5	28.3 ± 4.0***
18:0	8.5 ± 1.0	10.3 ± 1.3	10.8 ± 1.2	10.4 ± 1.6	14.4 ± 3.8	10.7 ± 0.9
Total saturate	30.2	41.9	18.8	24.0	22.3	44.7
16:1n-9	0.7 ± 0.2	0.7 ± 0.1	1.3 ± 0.3	3.7 ± 0.6*	0.3 ± 0.1	0.9 ± 0.2**
16:1n-7	0.6 ± 0.1	6.4 ± 0.9**	0.4 ± 0.2	3.8 ± 0.7**	1.2 ± 0.2	5.3 ± 1.2**
18:1n-9	34.9 ± 4.5	20.8 ± 1.1*	25.5 ± 1.5	24.5 ± 0.9	21.8 ± 3.2	17.1 ± 4.1
18:1n-7	2.8 ± 0.7	16.1 ± 2.3**	2.1 ± 0.6	10.7 ± 2.8*	2.7 ± 0.9	17.4 ± 3.0***
Total monoenoic	39.1	44.0	29.3	42.7	26.0	40.7
18:2n-6	11.2 ± 2.9	3.3 ± 0.9*	10.9 ± 2.1	6.6 ± 0.2*	15.6 ± 3.8	4.6 ± 1.1**
18:3n-6	12.4 ± 2.7	0.7 ± 0.3**	32.6 ± 0.9	19.0 ± 2.4***	29.1 ± 5.0	2.4 ± 1.9***
20:3n-6	4.5 ± 1.1	2.3 ± 0.2	3.5 ± 0.9	2.4 ± 1.2	3.6 ± 0.9	2.3 ± 0.8
20:4n-6	0.8 ± 0.1	3.1 ± 0.8*	0.3 ± 0.1	1.5 ± 0.1**	0.4 ± 0.1	2.4 ± 1.4*
Total n-6	28.9	9.4	47.3	29.5	48.7	11.7
18:3n-3	nd	nd	nd	nd	nd	nd
20:5n-3	nd	0.5 ± 0.3*	nd	nd	nd	nd
22:5n-3	1.3 ± 0.2	1.9 ± 0.5	1.6 ± 0.5	1.7 ± 0.1	1.2 ± 0.7	1.4 ± 0.6
22:6n-3	0.7 ± 0.1	2.3 ± 1.1	3.0 ± 0.6	2.1 ± 0.9	1.8 ± 0.6	1.5 ± 0.6
Total n-3	1.8	4.7	4.6	3.8	3.0	2.9

Fatty acid composition was determined by GC analysis as described in materials and methods. Data, expressed as mole percent, are means of 3 independent experiments ± SD

* *p* ≤ 0.05, ** *p* ≤ 0.01, *** p ≤ 0.001 significant differences between promastigote and amastigote by *t* test

**Table 2:** Fatty acid composition of total phospholipids, triacylglycerols and free fatty acid profile in *L. donovani* promastigote and amastigote

	Phospholipids		Triacylglycerols		Free fatty acids	
	Pro	Ama	Pro	Ama	Pro	Ama
14:0	0.6 ± 0.3	3.0 ± 0.5**	2.6 ± 0.8	4.0 ± 1.5	1.9 ± 0.7	4.0 ± 1.5
16:0	23.5 ± 3.2	26.5 ± 4.4	3.9 ± 0.9	10.5 ± 1.0**	6.9 ± 0.6	23.3 ± 0.9**
18:0	10.0 ± 1.7	9.7 ± 1.3	10.6 ± 1.2	10.0 ± 2.2	13.4 ± 1.9	13.0 ± 2.5
Total saturate	34.1	39.2	17.1	24.5	22.2	40.3
16:1n-9	0.5 ± 0.3	0.9 ± 0.3	1.5 ± 0.8	3.4 ± 0.7*	0.5 ± 0.2	1.2 ± 0.5
16:1n-7	0.7 ± 0.3	8.0 ± 0.8***	0.5 ± 0.2	4.1 ± 0.8**	1.3 ± 0.5	7.0 ± 2.4*
18:1n-9	35.9 ± 2.1	20.1 ± 0.2***	29.6 ± 3.4	22.1 ± 3.6	20.2 ± 3.7	19.3 ± 0.6
18:1n-7	3.3 ± 1.1	18.3 ± 2.3***	1.6 ± 0.2	13.8 ± 4.0*	2.6 ± 1.0	20.5 ± 4.3**
Total monoenoic	40.4	47.3	33.2	43.4	24.6	48.0
18:2n-6	9.5 ± 1.2	3.4 ± 1.0*	8.6 ± 2.5	6.4 ± 1.0	13.4 ± 1.7	3.7 ± 1.4*
18:3n-6	10.6 ± 3.0	0.6 ± 0.3**	31.8 ± 1.5	15.3 ± 2.4***	31.3 ± 3.8	1.3 ± 1.0***
20:3n-6	3.1 ± 1.4	1.5 ± 0.5	3.5 ± 0.9	3.5 ± 0.1	4.6 ± 0.2	1.1 ± 0.5*
20:4n-6	0.7 ± 0.2	3.5 ± 1.0**	0.3 ± 0.1	2.1 ± 0.8*	0.5 ± 0.1	2.3 ± 0.8*
Total n-6	23.9	9.3	44.2	27.3	49.5	8.4
18:3n-3	nd	nd	nd	nd	nd	nd
20:5n-3	nd	0.7 ± 0.3*	nd	nd	nd	nd
22:5n-3	1.1 ± 0.9	1.6 ± 0.4	1.9 ± 0.3	1.9 ± 0.1	1.4 ± 0.9	1.7 ± 0.2
22:6n-3	0.5 ± 0.1	1.7 ± 0.6	3.6 ± 0.8	2.9 ± 0.3	2.0 ± 0.8	1.6 ± 0.1
Total n-3	1.6	4.2	5.5	4.8	3.4	3.3

Fatty acid composition was determined by GC analysis as described in materials and methods. Data, expressed as mole percent, are means of 3 independent experiments ± SD

* *p* ≤ 0.05, ** *p* ≤ 0.01, *** *p* ≤ 0.001 significant differences between promastigote and amastigote by *t* test

PL from promastigotes contained primarily saturated and monounsaturated fatty acids whereas n-6 fatty acids were prominent in TG. For both lipids, n-6 fatty acids were found in much higher proportion than n-3 fatty acids, with n-6/n-3 ratios of 13.6 and 9.1 in PL and TG, respectively. Noticeably, both lipids showed high proportions of 18:2n-6/18:3n-6 and low proportion of 20:4n-6. The opposite was observed for n-3 fatty acids with higher proportions of the elongation/desaturation products 22:5n-3/22:6n-3 as compared to their 18C precursors (not detected). One may also notice that the distribution of fatty acids in the unesterified pool was very close to that of TG, which may suggest metabolic links for TG deacylation/reacylation pathway.

Important changes were observed in amastigotes (Tables 1, 2). Regarding PL, the monounsaturated fatty acid content was modified with a significant increase of n-7 fatty acids at the expense of 18:1n-9. Overall, the total n-6 fatty acids were decreased in PL. However, the proportion of 20:4n-6 was significantly increased by 5-fold, whereas the precursors 18:2 and 18:3n-6 were decreased. Conversely, n-3 fatty acids were increased, especially the end product 22:6n-3. These changes are reflected by a 5-fold higher n-3/n-6 ratio. Several of the changes are also observed in TG and free fatty acids. Especially, n-7 fatty acids and 20:4n-6 were highly increased, whereas n-9 fatty acid and n-6 precursors were decreased. In contrast to PL, no major changes were observed for n-3 fatty acids in TG and free fatty acids.

Table 3 summarizes these changes expressed as amastigote/promastigote ratios for the major lipid classes.

**Table 3:** Fatty acid change ratios in major lipids

	Phospholipids		Triacylglycerols		Free fatty acids	
	*L. infantum*	*L. donovani*	*L. infantum*	*L. donovani*	*L. infantum*	*L. donovani*
Saturated						
14:0	8.0	5.0	1.3	1.5	3.8	2.1
16:0	1.3	1.1	1.8	2.7	4.4	3.4
18:0	1.2	1.0	1.0	0.9	0.7	1.0
Monounsaturated						
16:1n-9	1.0	1.8	2.8	2.3	3.0	2.4
16:1n-7	10.7	11.4	9.5	8.2	4.4	5.4
18:1n-9	0.6	0.6	1.0	0.7	0.8	1.0
18:1n-7	5.8	5.5	5.1	8.6	6.4	7.9
n-6 polyunsaturated						
18:2n-6	0.3	0.4	0.6	0.7	0.3	0.3
18:3n-6	0.1	0.1	0.6	0.5	0.1	0.1
20:3n-6	0.5	0.5	0.7	1.0	0.6	0.2
20:4n-6	3.9	5.0	5.0	7.0	6.0	4.6
n-3 polyunsaturated						
22:5n-3	1.5	1.5	1.1	1.0	1.2	1.2
22:6n-3	3.3	3.4	0.7	0.8	0.8	0.8

Values represent the ratios Amastigote/Promastigote

## Discussion

The main objective of this study was to highlight major changes between promastigote and amastigote stages in terms of lipid distribution and fatty acid composition. We chose to purify the amastigotes from their host cells rather than to use *in vitro* axenic amastigotes, in order to mimic physiological conditions of the parasite life cycle, i.e. the internalization of the promastigotes by the macrophages and their subsequent transformation and multiplication. In this respect, our purification method provided an efficient yield and electronic microscopy showed a normal morphology of the purified amastigotes even if some of them suffered from the purification process.

With respect to lipid distribution, the hallmark of differentiation of promastigotes into amastigotes is the significant change of the cholesterol/PL ratio consequent to the 2-fold increase of cholesterol content and moderate decrease of PL content. This may result in major changes in surface membrane composition, although intracellular accumulation of cholesterol in the parasitophorous and in the cytoplasmic vacuoles cannot be excluded (Fig. 1). Regarding that host membrane cholesterol has been shown to be required for leishmanial infection based on cholesterol depletion with cyclodextrins [16], one may hypothesize that amastigote cholesterol originates from host membrane cholesterol. Recently, Yao and Wilson [17], showed that the sterol content of *Leishmania* promastigotes varies according to the changes in the culture medium and to all the changes that occur during metacyclogenesis. According to these authors these variations are due to the adsorption of cholesterol from the environment and at the same time to a decrease in the synthesis of ergosterol by the parasite.

A similar mechanism may be involved during differentiation from promastigote to amastigote. Our data also revealed a significant increase of the free fatty acid pool that may result from PL and TG hydrolysis.

The important role of cholesterol in the pathogenicity of visceral leishmaniasis has been widely studied and confirmed. Ghosh *et al*. in 2011 [18] found that a decrease in the serum cholesterol content of patients with VL was inversely proportional to the parasitic load in the spleen. In 2014, in another work, Ghosh *et al*. [19] reported that a decline in the level of membrane cholesterol during the infection by *Leishmania* disrupted membrane rafts.

Studies performed on hypercholesterolemic mice showed that the cholesterol level was directly related to the severity of disease progression [20]. Ghosh *et al*. [21] reported the requirement of host membrane cholesterol in the binding and internalization of *L. donovani* into macrophages using complementary approaches. They showed that treatment of macrophages in culture with the cholesterol carrier methyl ß-cyclodextrin (MbCD) resulted in specific removal of membrane cholesterol, and a concomitant reduction in binding and subsequent infection by *Leishmania* promastigotes [22]. We also observed a similar increase in cholesterol in axenic amastigotes, compared to promastigotes (results not shown) and studies on its origin and its role in ergosterol synthesis are currently under way.

In agreement with previous reports for several *Leishmania* species, including *L. donovani* [8, 23–25], the major PL in *L. donovani* and *L. infantum* promastigotes were found to be PtdCho and PtdEtn. Our results are also consistent with the literature with respect to lower content of PtdIns, PtdSer and no or only trace amounts of cardiolipin, phosphatidylglycerol and phosphatidic acid. The low proportion of CerPCho has also been reported, consistent with the fact that *Leishmania* parasites do not synthesize this lipid [8]. This suggests that amastigotes in which CerPCho was found in high proportion acquire this lipid from host cells. Interestingly, it was previously shown that the parasite could degrade CerPCho from host cells to increase its virulence [26]. The increase in PS in amastigotes may be related to studies indicating that in *Leishmania,* PS exposure may play a role in parasite apoptosis and the pathogenicity of leishmaniasis [8].

Fatty acid analyses of promastigote lipids from the two *Leishmania* species indicate higher proportions of unsaturated fatty acids (up to 6 unsaturations) compared to saturated ones. In PL, TG and free fatty acid pools, C18 fatty acids were predominant, including both saturated, mono and polyunsaturated ones. The long chain polyunsaturated fatty acids arachidonic and docosahexaenoic acids were found in low proportions. Overall, these observations are consistent with previous reports on lipid fatty acid composition in various *Leishmania* species [13, 23, 24, 27, 28]. Yet, several differences were noticeable, especially the absence of 18:3n-3 in all analyzed lipids, whereas this fatty acid was previously reported to be abundant in phosphatidylcholine from *Leishmania* promastigotes [24], but only minor in total phospholipids according to another study [29]. Moreover, other fatty acids reported before in lipids from *Leishmania* are not listed in the present data, as they were in low proportion and were not changed upon the differentiation process [13, 30]. Our data show a predominance of n-6 fatty acids in total PL. Of note, according to Adosraku *et al*. [25] distribution may vary among PL class, since they reported that PE is highly enriched in n-6 fatty acids whereas PC contains more n-3 fatty acids. Beach *et al*. [30] reported high content of docosahexaenoic acid in diphosphatidylglycerol. It has been previously proposed that these differences could be due to culture conditions, the age of the culture and the origin of the *Leishmania* strains [25].

Previous studies reported that both ∆6 and ∆5 elongases and ∆6, ∆5 and ∆4 desaturases were expressed in *Leishmania major* for the complete pathway of polyunsaturated fatty acid biosynthesis [1, 31]. Accordingly, both n-3 and n-6 elongation/desaturation products were detected in both *L. donovani* and *L. infantum*.

In promastigotes, n-6 fatty acids were present in a much higher proportion than n-3 fatty acids regardless of the lipid class, with a n-3/n-6 ratio between 0.07 and 0.10. Of note, in all lipid classes, the end-product 20:4n-6 was minor compared to the precursors 18:2 and 18:3n-6. Although ∆5 and ∆4 desaturases have been identified in *Leishmania* species, no 22C-n-6 fatty acids were detected. The opposite was observed for n-3 fatty acids, with the recovery of the end products 22:5n-3 and 22:6n-3, whereas the 18C and 20C-precursors were not detected. Interestingly, 22:6n-3 was found in equal or higher proportion than 20:4n-6 which is different from the plasma of their mammalian hosts, suggesting that these parasites closely regulate their FA composition.

Among the changes observed in amastigotes are the increase in total saturated and monounsaturated fatty acids and the decrease in total n-6 fatty acids regardless of the lipid class. The increase of saturated fatty acids is due to the specific increase of 16:0 for saturated FA, 18:1n-7 for monounsaturated FA and 20:4n-6 for n-6 FA. Conversely, n-6 precursors 18:2 and 18:3n-6 were specifically decreased. Of note, the fatty acid lipid composition is close to that of host cells (not shown), suggesting that the remodeling of lipids in amastigotes depends on fatty acids and/or on desaturase/elongase activities that are available in macrophages. The increase in 20:4n-6 may also suggest that these enzymes could be more efficient in amastigotes than in promastigotes, although long-chain n-3 fatty acids were not increased. It would be of interest to study the oxygenated fatty acid metabolism in *Leishmania* since eicosanoids derived from 20:4n-6 are well known to exert many biological activities in mammalian cells. Mi-Ichi *et al*. [32] reported that serum-derived fatty acids are essential for the intraerythrocytic proliferation of *Plasmodium falciparum* in humans. They showed that combinations of palmitic acid (16:0)/oleic acid (18:1 n-9), palmitic acid (16:0)/vaccenic acid (18:1 n-7), or stearic acid (18:0), promoted the intraerythrocytic parasite growth and they confirmed the importance of 16:0 and 18:1 n-9 during the erythrocytic cycle. Thus, 18:1 n-9 is mandatory for the intraerythrocytic proliferation of *P. falciparum*. Phospholipids are believed to be critical to virulence and viability in *Leishmania* [8]. As components of phospholipids and glycosylphosphatidylinositol anchors, fatty acids are responsible for forming the core of biological membranes and the correct localization of proteins within membranes [1]. The high phosphorylation dynamics during promastigote to amastigote differentiation [33] also suggests that protein phosphorylation cascades are involved in the developmental regulation of *Leishmania* parasites. Fatty acids can be used as energy sources and serve as signalling molecules or precursors for their synthesis. They also contribute to the anchoring of proteins by direct acylation of specific amino acids.

Our intracellular amastigotes model, together with the use of axenic amastigotes as an alternative model is useful for studying the host-cell infection by *Leishmania*. Since it is closer to the reality of the host-parasite interactions, it will help to elucidate the relationships between the host cholesterol and the parasite’s ergosterol and especially the role of cholesterol in both the transformation process and the sterol metabolism of *Leishmania*. Among the changes observed in amastigotes are the increase in total saturated and monounsaturated fatty acids and the decrease in total n-6 fatty acids, regardless of the lipid class. These changes might be of interest to study the specific role of some fatty acids in the virulence of *Leishmania.*

## Abbreviations

Ama: Amastigote
BSTFA: Bis(trimethylsilyl) trifluoroacetamide
FA: Fatty acid
FAME: Fatty acid methyl esters
CerPCho: Sphingomyelin
C: Total cholesterol
ERG: Ergosterol
GC: Gas chromatography
HILIC: Hydrophilic interaction liquid chromatography
LPtdCho: Lysophosphatidylcholine
LPtdEtn: Lysophosphatidylethanolamine
MbCD: Methyl-b-cyclodextrin
PBS: Phosphate-buffered saline
PL: Phospholipids
PLFA: Phospholipid fatty acid
Pro: Promastigote
PtdCho: Phosphatidylcholine
PtdEtn: Phosphatidylethanolamine
PtdIns: Phosphatidylinositol
PtdSer: Phosphatidylserine
SEM: Standard error of the mean
TG: Triglycerides
VL: Visceral leishmaniasis
